# Artemisia Ordosica Polysaccharides Enhance Antioxidant Capacity of Peripheral Blood Lymphocytes in Poultry Through Nrf2/Keap1 and TLR4/NF-κB Signal Pathway

**DOI:** 10.3390/antiox13111308

**Published:** 2024-10-28

**Authors:** Yuanyuan Xing, Yankai Zheng, Lu Chen, Yuanqing Xu, Xiao Jin, Li Hong, Sumei Yan, Binlin Shi

**Affiliations:** 1 College of Animal Science, Inner Mongolia Agricultural University, Hohhot 010018, China; xingyuanyuan2014@163.com (Y.X.); 13848911743@163.com (Y.Z.); xuyuanqing@imau.edu.cn (Y.X.); yaojinxiao@aliyun.com (X.J.); hl1116@imau.edu.cn (L.H.); yansmimau@163.com (S.Y.); 2 Animal Husbandry and Veterinary Department, Shanxi Animal Husbandry and Veterinary School, Taiyuan 030024, China; 17835115282@163.com

**Keywords:** Artemisia ordosica polysaccharides, antioxidant capacity, Nrf2/Keap1, TLR4/NF-κB

## Abstract

Artemisia ordosica polysaccharides (AOP) can promote animal growth, improve intestinal morphology, regulate immunity, and enhance antioxidant capacity. To investigate the antioxidant capacity of AOP, three experiments were conducted. (1) Different concentrations of AOP (0, 50, 100, 150, 200, and 250 μg/mL) and 1 µg/mL VA on peripheral blood lymphocytes (PBLs) treated with/without lipopolysaccharides (LPS) were investigated to select the optimum concentration. The results showed that 150 μg/mL AOP had significant antioxidation activity. (2) The PBLs was randomly divided into eight treatments with six replicates, namely CON, AOP, LPS, ML385 (Nrf2 inhibitor), AOP + LPS, AOP + ML385, LPS + ML385 and LPS + ML385 + AOP. The results showed that under a normal condition or stress condition, AOP presented antioxidation activity via upregulating Nrf2/Keap1 pathway-related gene expression. (3) The PBLs was randomly divided into eight treatments with six replicates, namely CON, AOP, LPS, PDTC (NF-κB inhibitor), AOP + LPS, AOP + PDTC, LPS + PDTC and LPS + PDTC + AOP. The results showed that under a normal condition, AOP presented antioxidation activity via increasing TLR4/NF-κB pathway-related gene expression; under a stress condition, AOP alleviated oxidative damage caused by LPS via suppressing TLR4/NF-κB pathway-related gene expression.

## 1. Introduction

Intensive animal husbandry frequently exposes livestock to oxidative stress from various stressors [1]. To meet the increased energy and nutrient demands under stress, livestock mobilize all the available defense mechanisms, which can lead to decreased immunity, reduced productivity, and even death [2]. Oxidative stress occurs when the production of free radicals exceeds the scavenging capacity, leading to an imbalance between oxidative and antioxidant processes [3]. The antioxidant system comprises enzymatic and non-enzymatic components. The enzymatic antioxidant system primarily includes endogenous enzymes such as superoxidase dismutase (SOD), catalase (CAT), and glutathione peroxidase (GPx), which constitute the first line of defense against oxidative damage. The expression of CAT, SOD, and GPx is mainly regulated by nuclear factor-erythroid 2-related factor 2 (Nrf2) [4]. Under normal conditions, Nrf2 is bound to Kelch-like ECH-associated protein 1 (Keap1) in the cytoplasm and remains inactive. However, when cells experience stress, Nrf2 dissociates from Keap1 and translocates to the nucleus [5]. Studies indicate that polysaccharides may mitigate oxidative stress in animals by activating the Nrf2/Keap1 signaling pathway, which enhances the activity of antioxidant enzymes and scavenges excess free radicals [6,7]. Additionally, polysaccharides have shown various bioactivities, including antinociceptive, anti-inflammatory, and antitumor effects, as well as potential for modulating the gut microbiota [8,9,10]. Therefore, polysaccharides are widely used in cancer treatment due to their safety, high efficacy, low toxicity, and minimal residue. They are also extensively utilized in the materials, food, and pharmaceutical industries [11]. However, their application in animal husbandry still demands extensive research and development.

Artemisia ordosica, commonly known as Ordos wormwood or Mongolian wormwood, is rich in nutrients such as proteins, fats, vitamins, and trace elements, and it also contains bioactive compounds, including polysaccharides, flavonoids, volatile oils, and triterpenoids [12]. Artemisia ordosica and its extracts play crucial roles in promoting animal growth, enhancing intestinal health, regulating immunity, and exerting antioxidative effects [12,13,14]. Polysaccharides, as the primary active components in Artemisia ordosica, have also demonstrated various biological functions in our previous studies, such as promoting the growth of broiler chickens, improving intestinal morphology, and enhancing the activity of intestinal digestive enzymes [15,16,17]. Based on our results, we hypothesize that these beneficial effects may be related to the regulation of the Nrf2/Keap1 and TLR4/NF-κB signaling pathways by AOP. To further validate this hypothesis, we evaluated the effects of varying concentrations of AOP on antioxidant indices, including the SOD, GPx, CAT, T-AOC (total antioxidant capacity), MDA (malondialdehyde), PC (protein carbonyl), ROS (reactive oxygen species), and 8-OHdG (8-hydroxy-2′-deoxyguanosine) of broiler peripheral blood lymphocytes (PBLs). We also examined the expression of genes associated with the Nrf2 and NF-κB signaling pathways, aiming to elucidate the mitigating effects of Artemisia ordosica polysaccharides (AOP) on oxidative stress in PBLs and determine the optimal concentration of AOP for subsequent in vitro experiments. As mentioned previously, the TLR4/NF-κB and Nrf2/Keap1 signaling pathways may be simultaneously involved in the regulation of oxidative stress by AOP in broilers. To explore this intersection and how AOP simultaneously regulates both TLR4/NF-κB and Nrf2/Keap1 signaling pathways, we used ML385, an inhibitor of the Nrf2/Keap1 pathway [18], and PDTC, an inhibitor of the TLR4/NF-κB pathway [19]. These inhibitors helped us investigate the interactions between these pathways and identify the points of convergence where AOP exerts its regulatory effects.

## 2. Materials and Methods

### 2.1. Preparation of AOP

Artemisia ordosica was collected from Erdos (Inner Mongolia, China, 39.82° N and 109.99° E) in July. The AOP were prepared using the method described by Xing et al. (2020) [15] and purified using DEAE-52 anion exchange column chromatography (Solarbio, Beijing, China, Cat. NO. 9013-34-7) and Sephadex G-100 gel column chromatography (Solarbio, Beijing, China, Cat. NO. 9050-94-6). The yields of purified polysaccharides were 29.32% of the total AOP, with a total sugar content of 88.79%. AOP with an average molecular weight of 9.00 kDa are a neutral polysaccharide composed of several monosaccharides, including fucose, arabinose, galactose, glucose, xylose, mannose, ribose, galacturonic acid, and glucuronic acid, with molar ratios of 0.56:13.75:12.79:54.08:3.15:13.43:0.63:0.67:0.93, respectively. AOP predominantly consist of glucosyl residues linked in a main chain, along with arabinosyl, galactosyl, mannose, and galacturonic acid residues in various configurations and linkages. The molar ratios of these structural groups in AOP are 66.43:1.55:4.81:3.19:6.48:6.74:9.17:1.65, respectively.

### 2.2. Collection and Separation Cultivation of Peripheral Blood Lymphocytes

The collection and separation cultivation of peripheral blood lymphocytes (PBLs) followed the method described by Tariq (2015) [20]. Blood samples were collected from the wing veins of 42-day-old broiler chickens obtained from Arbor Acres Poultry Breeding Company (Beijing, China) using sodium heparin as an anticoagulant. Five milliliters of blood sample was placed in a centrifuge tube, and an equal volume of lymphocyte separation medium (TBD, Tianjin, China, Cat. NO. LTS1090C) was slowly added. After centrifugation (2500 rpm, 20 min), the PBLs were collected into a 15 mL centrifuge tube. After washing twice with PBS wash solution, the cells were suspended in RPMI-1640 medium (Gibco, Grand Island, NY, USA, Cat. NO. 11879020) contained 10% fetal bovine serum (Gibco, Grand Island, NY, USA, Cat. NO. A5669701) and 1% penicillin-streptomycin (Gibco, Grand Island, NY, USA, Cat. NO. 15140122). Cell counting was performed using trypan blue, and the cell concentration was adjusted to 2 × 10^6^ cells/mL. The adjusted cell suspension was seeded in a 24-well sterile culture plate (Corning, NY, USA, Cat. NO.3337), with 2 mL of PBLs suspension in each well.

### 2.3. Treatment of PBLs with Signaling Molecules Inhibitors

We first examined the antioxidant capacity of AOP on PBLs treated with/without LPS to select the optimum concentration. Different concentrations of AOP (0, 50, 100, 150, 200, and 250 μg/mL) and 1 µg/mL VA were added to the culture medium. The cells were then cultured for 24 h in a constant temperature incubator (37 °C and 5% CO_2_). Afterward, each treatment was further divided into two groups: one group with the addition of 10 μg/mL LPS (Sigma-Aldrich, St. Louis, MO, USA, Cat. NO. L2880) as the stress group, and the other as the non-stress group, with continued cultivation for 6 h. After cultivation, the culture supernatants from each well were collected into centrifuge tubes, centrifuged (2500 rpm, 10 min), and the upper supernatant was frozen at −20 °C for the determination of antioxidant indicators (SOD, GPx, CAT, T-AOC, MDA, 8-OHdG, PC, and ROS activity or content) in the culture medium. The cells were also collected, and the total RNA was extracted for the determination of gene expression related to the TLR4/NF-κB and Nrf2/Keap1 signaling pathways in PBLs.

Next, we examined whether AOP alleviate oxidative injure in PBLs by regulating the Nrf2 pathway. ML385 (MCE, Monmouth Junction, NJ, USA, Cat. NO. HY-100523), an inhibitor of Nrf2, was incubated with AOP and LPS. The cells were divided into eight groups: a control group (CON), an AOP group (AOP, 150 μg/mL for 0.5 h), an AOP with ML385 group (AOP + ML385, 5 μmol/mL ML385 were treated for 12 h, and then treated with 150 μg/mL AOP), an ML385 group (ML385, 5 μmol/mL for 12 h), an LPS group (LPS, 10 μg/mL for 6 h), an AOP with LPS group (AOP + LPS, 150 μg/mL AOP were pretreated for 24, and then treated with 10 μg/mL LPS for 6 h), an LPS with ML385 group (LPS + ML385, 5 μmol/mL ML385 were treated for 12 h, and then treated with 10 μg/mL LPS for 6 h), and an ML385, AOP with LPS group (LPS + ML385+ AOP; 5 μmol/mL ML385 and 150 μg/mL AOP were pretreated for 12 h or 6 h, respectively, and then treated with 10 μg/mL LPS for 6 h). The contents of antioxidant indicators (SOD, GPx, CAT, T-AOC, MDA, 8-OHdG, PC, and ROS activity or content) in the culture medium, as well as the expression of genes related to the NF-κB and Nrf2 pathways, were measured.

Finally, we examined whether AOP alleviate oxidative injure in PBLs by regulating the TLR4/NF-κB pathway. PDTC (Abcam, Cambridge, UK, Cat. NO. ab141406), an inhibitor of NF-κB p65, was incubated with AOP and LPS. The cells were divided into eight groups: a control group (CON), an AOP group (AOP, 150 μg/mL for 0.5 h), an AOP with PDTC group (AOP + PDTC, 10 μmol/mL FDTC were treated for 0.5 h, and then treated with 150 μg/mL AOP), an PDTC group (PDTC, 10 μmol/mL for 0.5 h), an LPS group (LPS, 10 μg/mL for 6 h), an AOP with LPS group (AOP + LPS, 150 μg/mL AOP were pretreated for 24, and then treated with 10 μg/mL LPS for 6 h), an LPS with PDTC group (LPS + PDTC, 10 μmol/mL FDTC were treated for 0.5 h, and then treated with 10 μg/mL LPS for 6 h), and an PDTC, AOP with LPS group (LPS + PDTC+ AOP; 10 μmol/mL PDTC and 150 μg/mL AOP were pretreated for 0.5 h or 6 h, respectively, and then treated with 10 μg/mL LPS for 6 h). The contents of antioxidant indicators (SOD, GPx, CAT, T-AOC, MDA, 8-OHdG, PC, and ROS activity or content) in the culture medium, as well as the expression of genes related to the NF-κB and Nrf2 pathways, were measured.

### 2.4. Cell Viability Measurement

The cell viability was determined using the CCK Kit (MCE, Monmouth Junction, NJ, USA, HY-K0301) method. The PBLs cultured in the above treatments were seeded in a 96-well culture plate. After the cell culture and treatment were completed, 10 μL of CCK-8 reagent was added to each culture well. The plate was then incubated for an additional 4 h in the incubator, and the absorbance values of each culture well were measured at a wavelength of 490 nm. The cell viability was calculated using the following formula: Cell Viability (%) = (OD value of the treatment group − OD value of the blank group)/(OD value of the control group − OD value of the blank group) × 100.

### 2.5. Assay of Antioxidant Indicators in Cell Sample

The activity or content of antioxidant indicators, encompassing SOD (Cat. NO. A001-3-2), GPx (Cat. NO. H545-1-2), CAT (Cat. NO. A007-1-1), T-AOC (Cat. NO. A015-2-1), MDA (Cat. NO. A003-1-2), PC (Cat. NO. A087-1-2), 8-OHdG (Cat. NO. H165-1-1) and ROS (Cat. NO. S0033) were determined using commercially available kits (Nanjing Jiancheng Bioengineering Institute, Nanjing, China), following the manufacturer’s instructions meticulously.

### 2.6. RNA Preparation and Fluorescence Quantitative Real-Time PCR

The total RNA extraction from the PBLs was performed using the TRIzol (Takara Bio Inc., Otsu, Japan, Cat. NO. 9109) extraction method, adhering to the manufacturer’s instructions. Quantitative and qualitative assessment of the RNA was carried out using an ultraviolet spectrophotometer (BioTek, Winooski, VT, USA) at 260 nm and 280 nm. The RNA integrity was evaluated via horizontal electrophoresis on 1.5% agarose gel (Solarbio, Beijing, China, Cat. NO. A8201). Subsequent cDNA synthesis was executed utilizing the PrimeScript RT Reagent Kit with gDNA Eraser (Takara Bio Inc., Otsu, Japan, Cat. NO. 6110A), following the manufacturer’s protocol. Quantitative real-time PCR (qPCR) was performed by employing TB Premix Ex Taq II (Takara Bio Inc., Otsu, Japan, Cat. NO. RR055A), as per the manufacturer’s instructions, on an Illumina real-time PCR machine(Illumina, San Diego, CA, USA). The procedure encompassed an initial denaturation cycle at 95 °C for 30 s, followed by 40 cycles comprising denaturation at 95 °C for 5 s and annealing at 60 °C for 30 s. Subsequently, a dissociation stage generated a melting curve to verify the specificity of the amplified products. The target genes, including toll-like receptor 4 (*TLR4*), myeloid differentiation primary response 88 (*MyD88*), inhibitor of NF-κB alpha (*IκBα*), inhibitory kappa B kinase beta (*IKKβ*), nuclear factor kappa-B p65 (*NF-κB p65*), interleukin-1β (*IL-1β*), interleukin-6 (*IL-6*), nuclear factor erythroid-2-related factor 2 (*Nrf2*), Kelch-like ECH-associated protein 1 (*keap1)*, *GPx*, *CAT*, *SOD*, along with their primer sequences, as in a previous study (Xing et al., 2023) [15], and designed by Shanghai Sangon Biotech (Shanghai, China). The primers designed for *IκBα* and *IKKβ* genes and sequences of amplicons are shown below: *IκBα*, accession no.: NM_001001472.2, F-GGCAGATGTGAACAAGGTGA and R-TATCTGCAGGTCAGCTGTGG; *IKKβ*, accession no.: XM_046903365.1, F-TGATAGCAAGGTGAATGACGCTGTAG and R-CGGATGAGGTCGCAAGGCAAC. The sizes of the PCR products were as follows: 118 bp for *IκBα* and 140 bp for *IKKβ*. β-actin served as the reference gene and was quantified using TB Green and the 2^−∆∆Ct^ method.

### 2.7. Data Processing and Statistical Analysis

Data were tested for the normality of the distribution (PROC UNIVARIATE) and the homogeneity of variance was tested using Levene’s homogeneity-of-variance test. Differences between treatment groups for normally distributed data were determined using one-way analysis of variance (ANOVA) followed by the Tukey–Kramer honest significant difference test using SAS (version 9.4, SAS Institute, Cary, NC, USA). All the graphs were drawn on GraphPad Prism (version 8.0, GraphPad Software, San Diego, CA, USA) and Photoshop software (version CS6, Adobe, San Jose, CA, USA). The results were expressed as the mean values ± standard error of the mean (SEM). A probability level of *p* < 0.05 was chosen as the limit for statistical significance.

## 3. Results

### 3.1. Effects of AOP on the Cell Viability of PBLs

**Figure 1:**
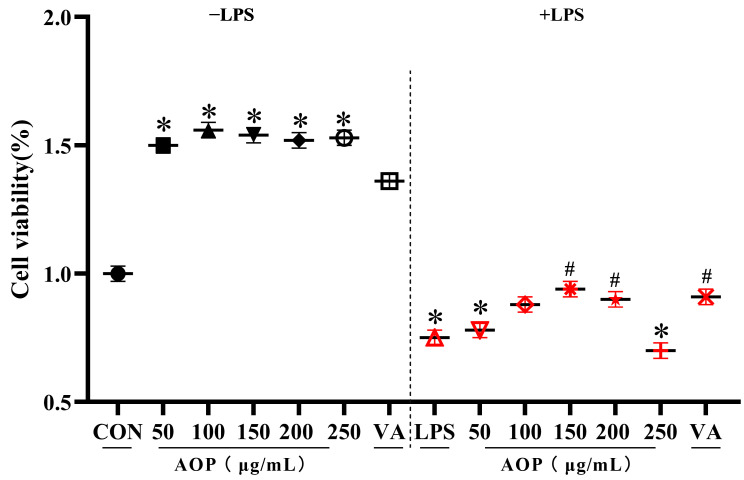
Effects of AOP on the cell viability of PBLs (%). Note: Different concentration of AOP (0, 50, 100, 150, 200, and 250 μg/mL) and 1 µg/mL VA were supplemented to the culture medium for 24 h. Afterward, each treatment was further divided into two groups: one group with the addition of 10 μg/mL LPS as the stress group, and the other as the non-stress group, with continued cultivation for 6 h. Each value is shown as the mean ± SEM (n = 6); * *p* < 0.05 or vs. control group; ^#^ *p* < 0.05 vs. LPS group.

As shown in Figure 1, under non-stress conditions, AOP at concentrations ranging from 50 to 250 μg/mL enhanced the PBLs activity, exceeding the effects observed in the VA treatment group.

Under stress conditions challenged by lipopolysaccharide (LPS), concentrations of 150 and 200 μg/mL of AOP significantly mitigated the LPS-challenged decrease in PBLs viability (*p* < 0.05). Moreover, the protective effect of AOP was comparable to that of VA.

### 3.2. Effects of AOP on Antioxidant Enzyme Activity and Oxidative Stress Metabolites in PBLs

**Figure 2:**
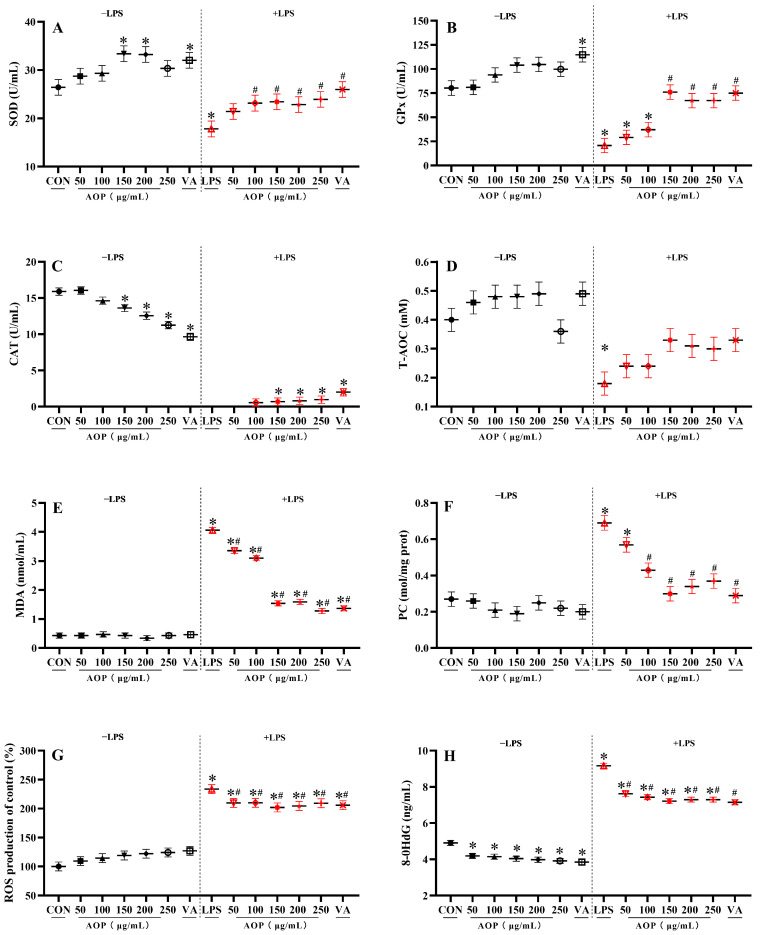
Effects of AOP on the antioxidant enzyme activity and oxidative stress metabolites in PBLs. Note: (**A**–**H**): SOD, superoxide dismutase; GPx, glutathione peroxidase; CAT, catalase; T-AOC, total antioxidant capacity; MDA, malondialdehyde; PC, protein carbonyl; ROS, reactive oxygen species; 8-OHdG, 8-hydroxy-2′-deoxyguanosine. Different concentrations of AOP (0, 50, 100, 150, 200, and 250 μg/mL) and 1 µg/mL VA were supplemented to the culture medium for 24 h. Afterward, each treatment was further divided into two groups: one group with the addition of 10 μg/mL LPS as the stress group, and the other as the non-stress group, with continued cultivation for 6 h. Each value is shown as the mean ± SEM (n = 6); * *p* < 0.05 or vs. control group; ^#^ *p* < 0.05 vs. LPS group.

As shown in Figure 2, under non-stress conditions, supplementation with 150–200 µg/mL of AOP significantly increased the SOD enzyme activity and decreased the 8-OHdG levels compared to the control group, with no significant difference observed compared to the VA treatment group (*p* < 0.05).

Under stress conditions challenged by LPS, AOP effectively mitigated the decline in antioxidant enzyme activity and reduced the excessive accumulation of oxidative stress metabolites in the culture medium of the PBLs. Specifically, compared to the control group, supplementation with 100–250 µg/mL AOP improved the SOD enzyme activity and reduced the PC content, showing protective effects comparable to those of VA (*p* < 0.05). Additionally, supplementation with 150–250 μg/mL AOP attenuated the decrease in the GPx and CAT enzyme activities and the increase in the MDA content, with no significant difference compared to the VA treatment group (*p* < 0.05). Moreover, AOP at concentrations ranging from 50 to 250 μg/mL effectively reduced the increase in the 8-OHdG and ROS content, with no significant difference observed between the AOP and VA treatment groups (*p* < 0.05).

### 3.3. Effects of AOP on Nrf2 Signaling Pathway-Related Gene Expression in PBLs

**Figure 3:**
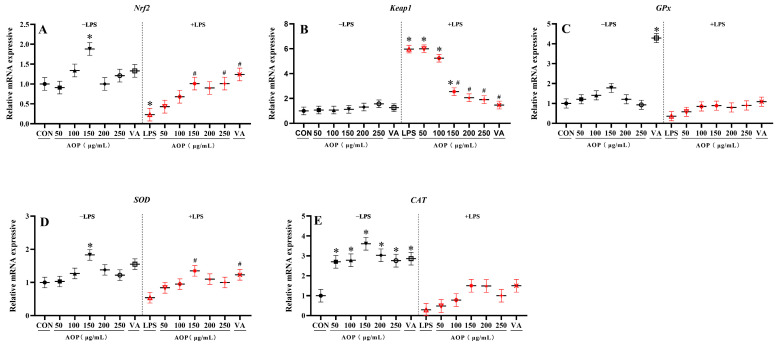
Effects of AOP on the Nrf2 signaling pathway-related gene expression in PBLs. Note: (**A**–**E**): *Nrf2*, nuclear factor erythroid-2-related factor 2; *Keap1*, Kelch-like ECH-associated protein 1; *GPx*, glutathione peroxidase; *SOD*, superoxide dismutase; *CAT*, catalase. Different concentrations of AOP (0, 50, 100, 150, 200, and 250 μg/mL) and 1 µg/mL VA were supplemented to the culture medium for 24 h. Afterward, each treatment was further divided into two groups: one group with the addition of 10 μg/mL LPS as the stress group, and the other as the non-stress group, with continued cultivation for 6 h. The gene expression for β-actin was used as a housekeeping gene. The relative expression levels from the control group were used as reference values. Each value is shown as the mean ± SEM (n = 6); * *p* < 0.05 or vs. control group; ^#^ *p* < 0.05 vs. LPS group.

As shown in Figure 3, under non-stress conditions, supplementation with various concentrations of AOP significantly enhanced the expression of the *CAT* gene compared to the control group, with no significant difference compared to the VA treatment group (*p* < 0.05). Additionally, 150 μg/mL AOP significantly increased the expression of the *Nrf2* and *SOD* genes, showing protective effects comparable to those of the VA treatment group (*p* < 0.05).

Under stress conditions challenged by LPS, different concentrations of AOP significantly alleviated the decrease in the expression of antioxidant enzyme-related genes in the culture medium of the PBLs, while also reducing the *Keap1* overexpression. Specifically, compared to the control group, supplementation with 150–250 μg/mL AOP mitigated the reduction in *Nrf2* and *SOD* gene expression and decreased the *Keap1* overexpression, with protective effects comparable to those observed with VA (*p* < 0.05).

### 3.4. Effects of AOP on NF-κB Signaling Pathway-Related Gene Expression in PBLs

**Figure 4:**
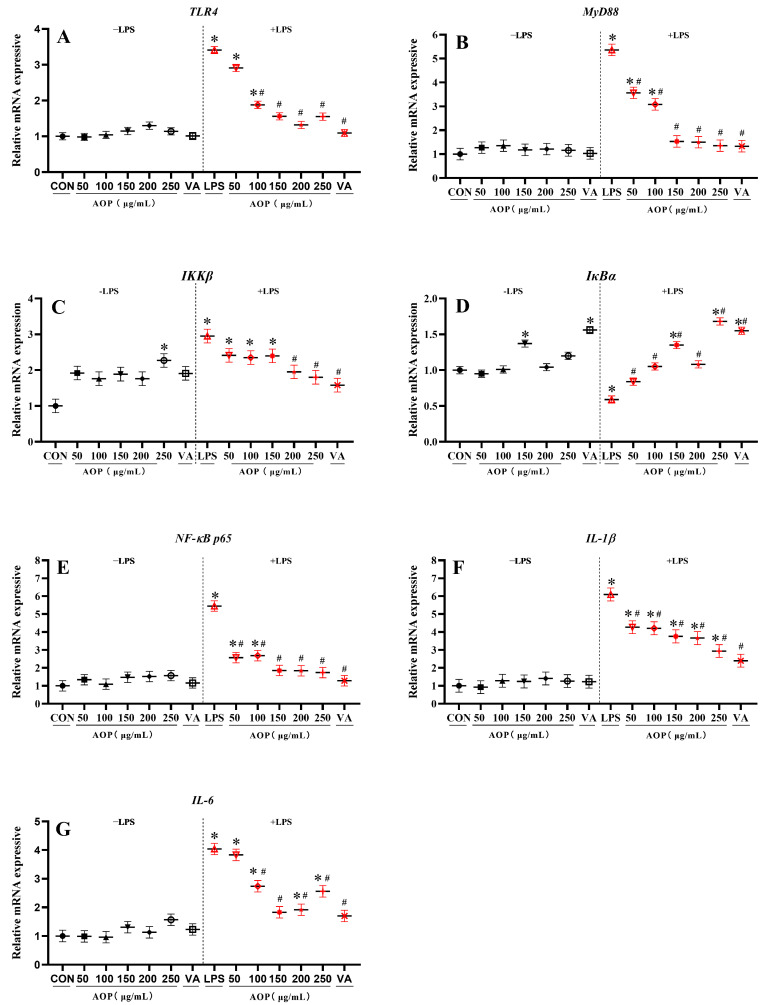
Effects of AOP on the NF-κB signaling pathway-related gene expression in PBLs. Note: (**A**–**G**): *TLR4*, toll-like receptor 4; *MyD88*, myeloid differentiation primary response 88; *IKKβ*, inhibitory kappa B kinase beta; *IκBα*, inhibitor of NF-κB alpha; *NF-κB p65*, nuclear factor kappa-B p65; *IL-1β*, interleukin-1β and *IL-6*, interleukin-6. Different concentrations of AOP (0, 50, 100, 150, 200, and 250 μg/mL) and 1 µg/mL VA were supplemented to the culture medium for 24 h. Afterward, each treatment was further divided into two groups: one group with the addition of 10 μg/mL LPS as the stress group, and the other as the non-stress group, with continued cultivation for 6 h. The gene expression for β-actin was used as a housekeeping gene. The relative expression levels from the control group were used as reference values. Each value is shown as the mean ± SEM (n = 6); * *p* < 0.05 or vs. control group; ^#^ *p* < 0.05 vs. LPS group.

As shown in Figure 4, under non-stress conditions, compared with the control group, 250 µg/mL AOP increased the gene expression of *IKKβ* in the PBLs, while 150 µg/mL AOP increased the gene expression of *IkBα* in the PBLs (*p* < 0.05). AOP did not significantly affect the expression of other genes of NF-κB signaling pathway-related factors in the PBLs.

Under stress conditions, adding various concentrations of AOP to the PBLs culture medium significantly reduced the LPS-challenged overexpression of NF-κB pathway-related genes in the PBLs. Compared to the control group, 100–250 µg/mL AOP significantly mitigated the LPS-challenged *TLR4* expression, with protective effects comparable to the VA-treated group (*p* < 0.05). Additionally, 150–250 µg/mL AOP significantly reduced the overexpression of the *IKKβ*, *MyD88*, *NF-κB P65*, *IL-1β*, and *IL-6* genes (*p* < 0.05), showing no significant difference from the VA-treated group. Additionally, different concentrations of AOP and VA significantly alleviated the reduction of *IkBα* in broiler PBLs caused by LPS (*p* < 0.05).

### 3.5. Effects of AOP on Antioxidative Enzymes and Oxidative Stress Metabolites in PBLs Challenged by LPS and Blocked by ML385

**Figure 5:**
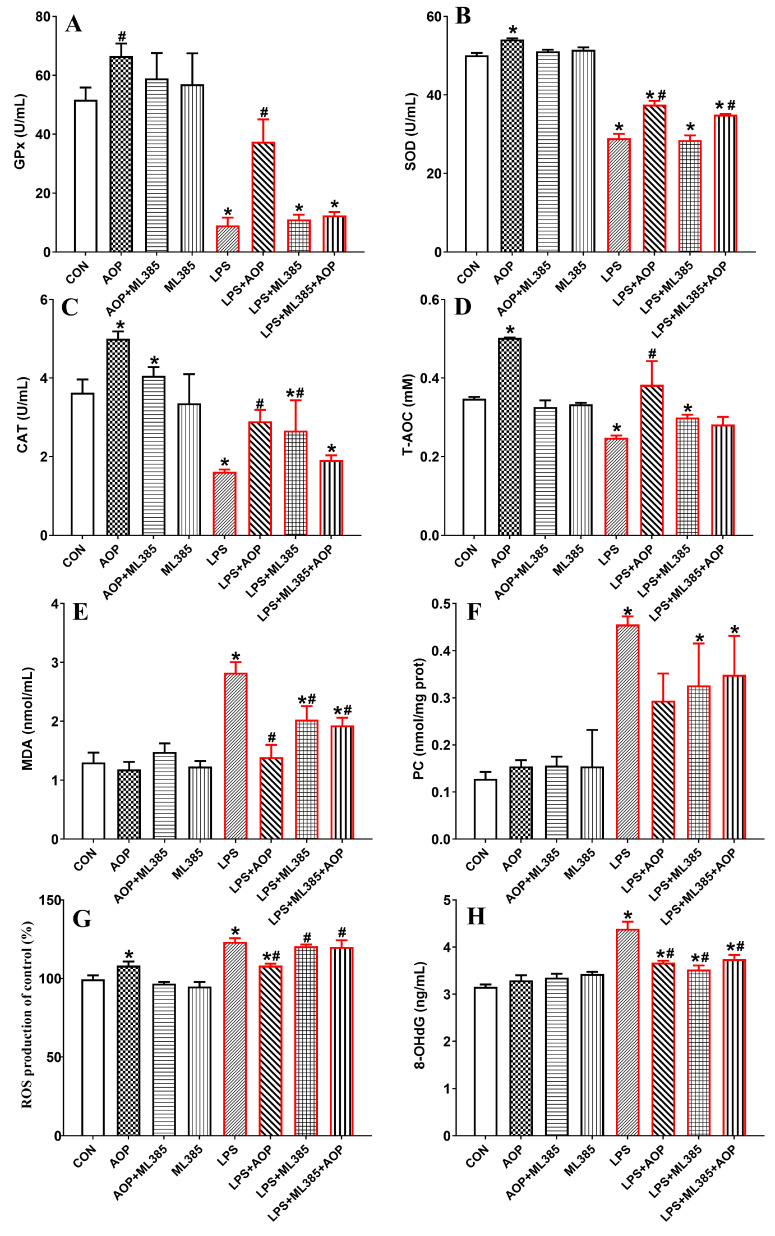
Effects of AOP on the antioxidative enzymes and oxidative stress metabolites in PBLs challenged by LPS and blocked by ML385. Note: (**A**–**H**): GPx, glutathione peroxidase; SOD, superoxide dismutase; CAT, catalase; T-AOC, total antioxidant capacity; MDA, malondialdehyde; PC, protein carbonyl; ROS, reactive oxygen species; 8-OHdG, 8-hydroxy-2′-deoxyguanosine. CON (control group), LPS (lipopolysaccharide), AOP (Artemisia ordosica polysaccharides), and ML385 (Nrf2 inhibitor). Each value is shown as the mean ± SEM (n = 6); * *p* < 0.05 or vs. control group; ^#^ *p* < 0.05 vs. LPS group.

As shown in Figure 5, the AOP-treated group exhibited increased T-AOC and activities of SOD and CAT in PBLs compared to the control group (CON). In contrast, the T-AOC and the activities of SOD and CAT in the AOP + ML385 group were significantly lower than those in the AOP group, with no significant changes observed in any indices in the ML385-treated group (*p* < 0.05).

Compared to the CON and AOP groups, the LPS stress group significantly increased the levels of MDA, 8-OHdG, ROS, and PC in the culture medium of the PBLs, and it significantly decreased the T-AOC, SOD, GPx, and CAT activities. However, the AOP + LPS group alleviated the LPS-challenged reduction in the antioxidant enzyme activities and the excessive production of oxidative stress products. In comparison to the AOP + LPS group, the ML385 + LPS and AOP + LPS + ML385 groups showed decreased T-AOC, SOD, and GPx activities in the PBLs, alongside increased levels of MDA and ROS (*p* < 0.05).

### 3.6. Effects of AOP on the Gene Expression of Nrf2 Signaling Pathway-Related Factors in PBLs Challenged by LPS and Blocked by ML385

**Figure 6:**
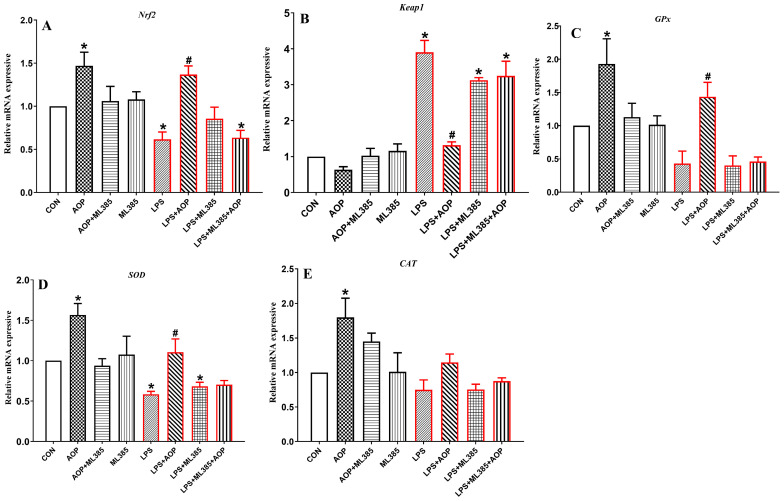
Effects of AOP on the gene expression of the Nrf2 signaling pathway in PBLs challenged by LPS and blocked by ML385. Note: (**A**–**E**): *Nrf2*, nuclear factor erythroid-2-related factor 2; *Keap1*, Kelch-like ECH-associated protein 1; *GPx*, glutathione peroxidase; *SOD*, superoxide dismutase; *CAT*, catalase. CON (control group), LPS (lipopolysaccharide), AOP (Artemisia ordosica polysaccharides), and ML385 (Nrf2 inhibitor). The gene expression for β-actin was used as a housekeeping gene. The relative expression levels from the control group were used as reference values. Each value is shown as the mean ± SEM (n = 6); * *p* < 0.05 or vs. control group; ^#^ *p* < 0.05 vs. LPS group.

As shown in Figure 6, the AOP-treated group increased the gene expression of *Nrf2* and its downstream target genes (*SOD*, *GPx*, and *CAT*) in the PBLs compared to the CON group. In the AOP + ML385 group, the expression of *Nrf2*, *SOD*, and *GPx* genes in the PBLs was lower than that in the AOP group, with no significant changes in any indices in the ML385-treated group (*p* < 0.05).

Compared to the CON and AOP groups, the LPS stress group significantly reduced the gene expression of *Nrf2* and its downstream target genes (*SOD*, *GPx*, and *CAT*) in the PBLs but promoted the overexpression of the Keap1 gene. However, the AOP + LPS group mitigated the LPS-challenged overexpression of Nrf2 and its downstream target genes (*SOD*, *GPx*, and *CAT*) while also reducing the overexpression of the Keap1 gene. Compared to the AOP + LPS group, the ML385 + LPS and AOP + LPS + ML385 groups decreased the gene expression of *Nrf2*, *SOD*, and *GPx* in the PBLs but increased the gene expression of Keap1 (*p* < 0.05).

### 3.7. Effects of AOP on the Gene Expression of NF-κB Signaling Pathway-Related Factors in PBLs Challenged by LPS and Blocked by ML385

**Figure 7:**
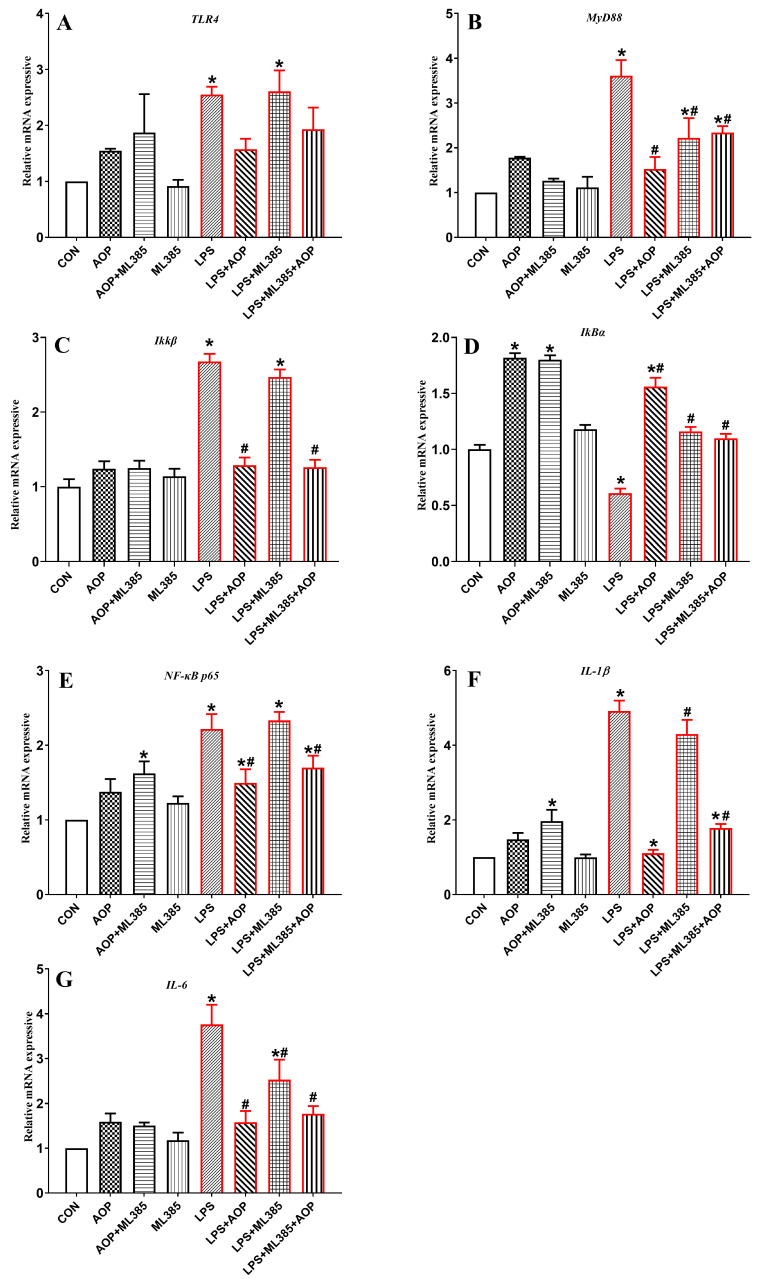
Effects of AOP on the gene expression of the NF-κB signaling pathway in PBLs challenged by LPS and blocked by ML385. Note: (**A**–**G**): *TLR4*, toll-like receptor 4; *MyD88*, myeloid differentiation primary response 88; *IKKβ*, inhibitory kappa B kinase beta; *IκBα*, inhibitor of NF-κB alpha; *NF-κB p65*, nuclear factor kappa-B p65; *IL-1β*, interleukin-1β; *IL-6*, interleukin-6. CON (control group), LPS (lipopolysaccharide), AOP (Artemisia ordosica polysaccharides), and ML385 (Nrf2 inhibitor). β-actin was used as a housekeeping gene. The relative expression levels from the control group were used as reference values. Each value is shown as the mean ± SEM (n = 6); * *p* < 0.05 or vs. control group; ^#^ *p* < 0.05 vs. LPS group.

As shown in Figure 7, no significant changes were observed in any of the indices in the ML385-treated group compared to the control group (CON). The AOP-treated group increased the gene expression of *IkBα*, *NF-κB p65* and *IL-1β* (*p* < 0.05).

Compared to the CON and AOP groups, the LPS group significantly increased the gene expression of factors associated with the NF-κB signaling pathway and reduced the gene expression of *IkBα*. The AOP + LPS and LPS + ML385 + AOP treatments both mitigated this overexpression challenged by LPS. However, no significant differences were observed between the AOP + LPS and LPS + ML385 + AOP treatment groups. Additionally, there were no significant differences between the ML385 + AOP group and the other treatment groups. The expression of the *MyD88* and *IL-6* genes was reduced in the LPS + ML385 group compared to the LPS stress group, but the expression levels of other inflammatory factor-related genes did not differ significantly from those in the LPS stress group (*p* < 0.05).

### 3.8. Effects of AOP on Antioxidative Enzymes and Oxidative Stress Metabolites in PBLs Challenged by LPS and Blocked by PDTC

**Figure 8:**
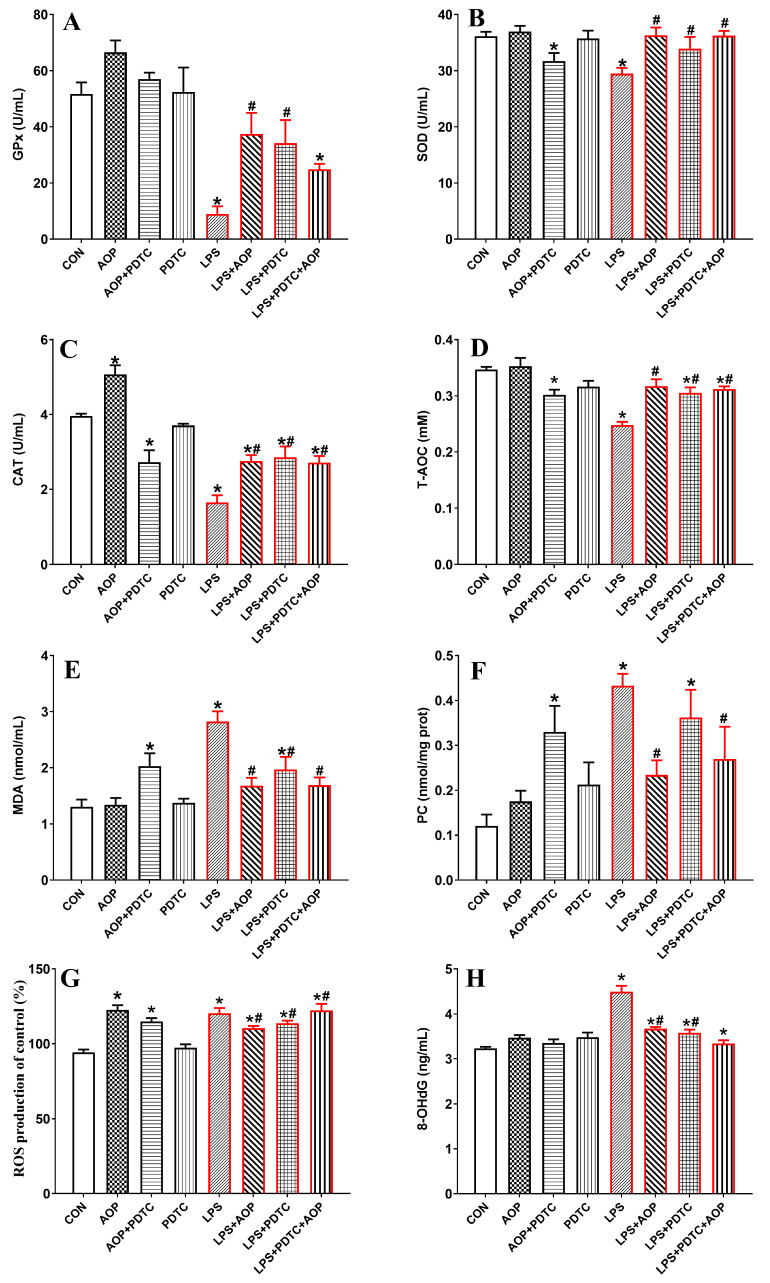
Effects of AOP on the antioxidative enzymes and oxidative stress metabolites in PBLs challenged by LPS and blocked by PDTC. Note: (**A**–**H**): GPx, glutathione peroxidase; SOD, superoxide dismutase; CAT, catalase; T-AOC, total antioxidant capacity; MDA, malondialdehyde; PC, protein carbonyl; ROS, reactive oxygen species; 8-OHdG, 8-hydroxy-2′-deoxyguanosine. CON (control group), LPS (lipopolysaccharide), AOP (Artemisia ordosica polysaccharides), and PDTC (NF-κB inhibitor). Each value is shown as the mean ± SEM (n = 6); * *p* < 0.05 or vs. control group; ^#^ *p* < 0.05 vs. LPS group.

As shown in Figure 8, the AOP-treated group showed increased CAT activity and ROS levels in the PBLs compared to the CON group (*p* < 0.05). However, the T-AOC, SOD, and CAT activities were significantly lower than those in the AOP group, while the MDA content was significantly higher than that in the AOP group (*p* < 0.05). No significant changes were observed in any of these indices in the PDTC-treated group (*p* > 0.05).

The LPS stress group significantly increased the MDA, 8-OHdG, ROS, and PC content in the culture medium of the PBLs compared to the CON and AOP groups (*p* < 0.05). It also significantly decreased the T-AOC, SOD, GPx, and CAT activities (*p* < 0.05). However, treatment with LPS in combination with AOP, PDTC, or both (LPS + AOP, LPS + PDTC, LPS + PDTC + AOP) mitigated the excessive decrease in the antioxidant enzyme activities and the overproduction of oxidative stress products challenged by LPS. Specifically, the LPS + PDTC + AOP group exhibited significantly lower ROS levels compared to the LPS + AOP and LPS + PDTC groups (*p* < 0.05).

### 3.9. Effects of AOP on the Gene Expression of Nrf2 Signaling Pathway-Related Factors in PBLs Challenged by LPS and Blocked by PDTC

**Figure 9:**
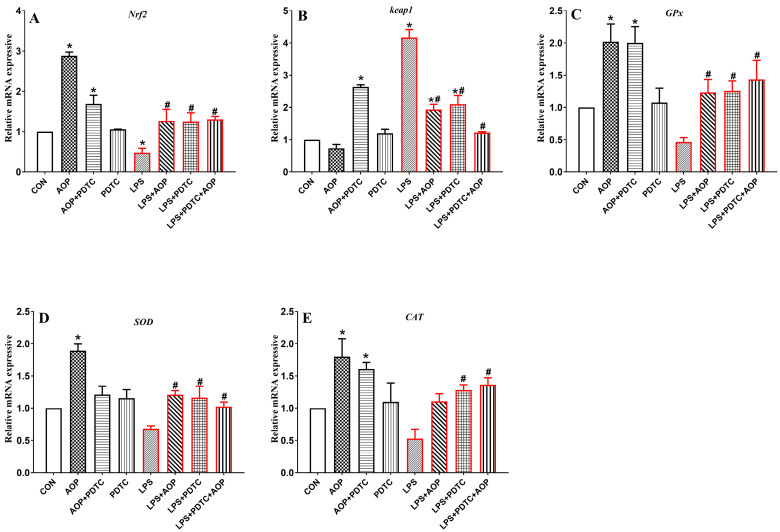
Effects of AOP on the gene expression of the Nrf2 Signaling pathway in PBLs challenged by LPS and blocked by PDTC. Note: (**A**–**E**): *Nrf2*, nuclear factor erythroid-2-related factor 2; *Keap1*, Kelch-like ECH-associated protein 1; *GPx*, glutathione peroxidase; *SOD*, superoxide dismutase; *CAT*, catalase. CON (control group), LPS (lipopolysaccharide), AOP (Artemisia ordosica polysaccharides), and PDTC (NF-κB inhibitor). β-actin was used as a housekeeping gene. The relative expression levels from the control group were used as reference values. Each value is shown as the mean ± SEM (n = 6); * *p* < 0.05 or vs. control group; ^#^ *p* < 0.05 vs. LPS group.

As shown in Figure 9, the AOP-treated group exhibited increased gene expression of Nrf2 and its downstream target genes (*SOD*, *GPx*, and *CAT*) in the PBLs compared to the control group (CON), along with decreased expression of Keap1 (*p* < 0.05). Conversely, the AOP + PDTC group showed lower levels of *Nrf2* and *SOD* gene expression compared to the AOP group, with higher *Keap1* gene expression (*p* < 0.05). No significant changes were observed in any of the indicators in the PDTC-treated group.

In the LPS stress group, the gene expression of Nrf2 and its downstream target genes (*SOD*, *GPx*, and *CAT*) was significantly reduced, while the *Keap1* gene expression was significantly increased compared to the CON and AOP groups (*p* < 0.05). The LPS + AOP, LPS + PDTC, and LPS + PDTC + AOP treatments mitigated the excessive decrease in *Nrf2* and its downstream target genes caused by LPS and reduced the overexpression of *Keap1* (*p* < 0.05). Notably, the LPS + PDTC + AOP group showed significantly lower *Keap1* gene expression compared to both the LPS + AOP and LPS + PDTC groups (*p* < 0.05).

### 3.10. Effects of AOP on the Gene Expression of NF-κB Signaling Pathway-Related Factors in PBLs

**Figure 10:**
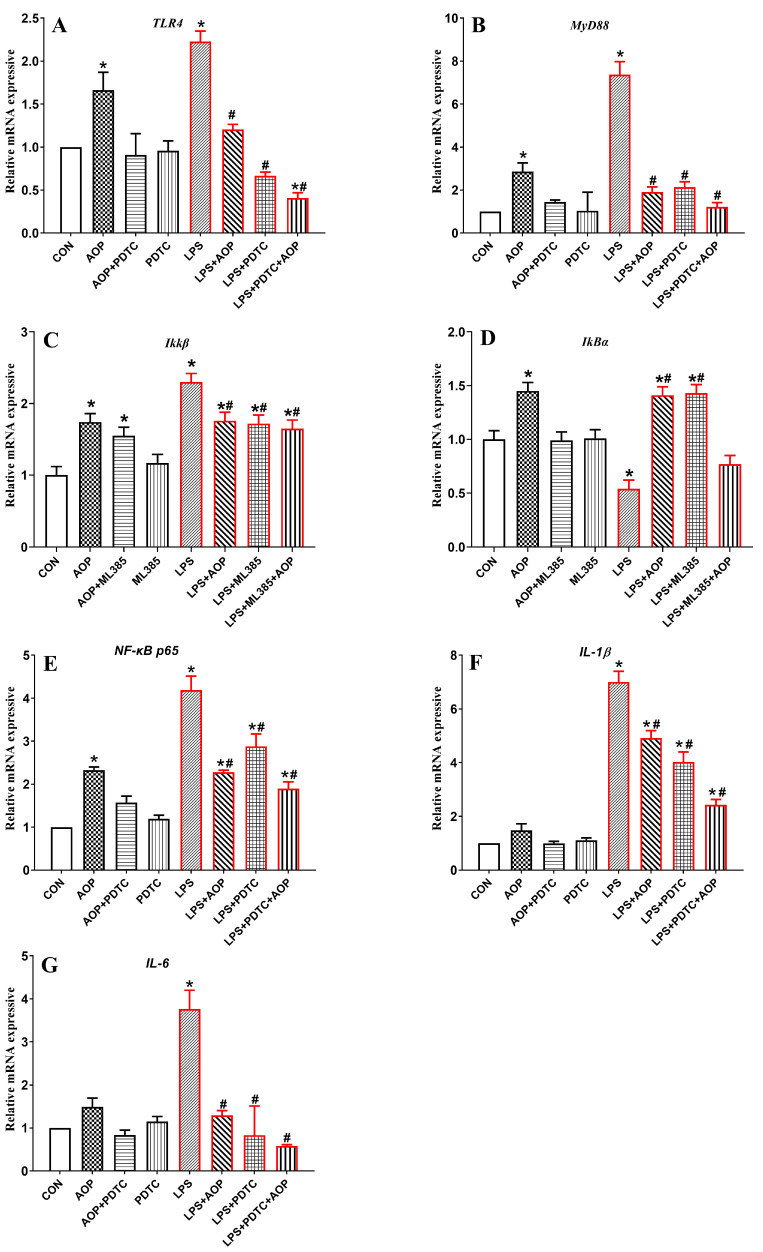
Effects of AOP on the gene expression of the NF-κB signaling pathway in PBLs challenged by LPS and blocked by PDTC. Note: (**A**–**G**): *TLR4*, toll-like receptor 4; *MyD88*, myeloid differentiation primary response 88; *IKKβ*, inhibitory kappa B kinase beta; *IκBα*, inhibitor of NF-κB alpha; *NF-κB p65*, nuclear factor kappa-B p65; *IL-1β*, interleukin-1β; *IL-6*, interleukin-6. CON (control group), LPS (lipopolysaccharide), AOP (Artemisia ordosica polysaccharides), and PDTC (NF-κB inhibitor). β-actin was used as a housekeeping gene. The relative expression levels from the control group were used as reference values. Each value is shown as the mean ± SEM (n = 6); * *p* < 0.05 or vs. control group; ^#^ *p* < 0.05 vs. LPS group.

As shown in Figure 10, the AOP-treated group exhibited increased expression of the *TLR4*, *MyD88*, *IkBα*, *IKKβ* and *NF-κB P65* genes (*p* < 0.05). In contrast, the AOP + PDTC group attenuated the upregulation of these genes challenged by AOP (*p* < 0.05), with no significant changes observed in any of the indices in the PDTC-treated group.

Compared to the CON and AOP groups, the LPS group significantly upregulated the gene expression of the NF-κB signaling pathway-related factors and decreased the expression of the *IkBα* gene (*p* < 0.05). However, treatment with LPS in combination with AOP, PDTC, or both (LPS + AOP, LPS + PDTC, LPS + PDTC + AOP) mitigated the overexpression of these genes challenged by LPS and increased the expression of the *IkBα* gene (*p* < 0.05). Specifically, the LPS + PDTC + AOP group showed no significant changes in all the indicators. The gene expression of the signaling pathway-related factors in the CON and AOP groups was lower than that in the LPS + AOP and LPS + PDTC groups (*p* < 0.05).

## 4. Discussion

### 4.1. Alleviating Effect of AOP on Oxidative Stress of PBLs

Oxidative stress is characterized by damage to the organism resulting from an excessive accumulation of free radicals, which occurs due to an imbalance between oxidation and antioxidation processes, either from increased radical generation or decreased scavenging capacity [21]. Studies have found that the protective effect of polysaccharides is mainly achieved through the activation of the Nrf2/Keap1 pathway, which increases the activity of antioxidant enzymes and, thereby, protects cells from oxidative damage [22,23]. In addition, polysaccharides can relieve the intracellular inflammatory environment by reducing the expression of inflammatory cytokines, possibly by inhibiting the NF-κB/TLR4 pathway [23,24]. In our current experiment, the addition of various concentrations of AOP to the culture medium of PBLs significantly mitigated the LPS-challenged decrease in antioxidant enzyme activities (SOD, GPx, and CAT) and reduced the levels of excessive oxidative stress metabolites (MDA, 8-OHdG, PC, and ROS). The optimal effect was observed with 150 µg/mL AOP, demonstrating a protective effect comparable to that of VA. This effect is likely due to the ability of AOP to counteract the LPS-challenged reduction in *Nrf2* expression and its downstream target genes while reducing the overexpression of *Keap1* and the TLR4/NF-κB signal pathway. Additionally, a previous study demonstrated that polysaccharides can mitigate oxidative damage by reducing the cellular morphological and structural damage and lowering the apoptotic rate [25,26]. This mechanism may be relevant to how AOP alleviates oxidative damage, although the specific pathways involved warrant further investigation.

Our study demonstrated that the supplementation of 150–200 µg/mL AOP significantly increased the SOD enzyme activity and reduced the 8-OHdG levels compared to the control group under non-stressed conditions. This was consistent with the observed increase in *Nrf2* and *SOD* gene expression at 150 µg/mL AOP. In conclusion, AOP enhances the antioxidant capacity of broiler PBLs by upregulating Nrf2 and its downstream targets while downregulating *Keap1* expression under both stressed and non-stressed conditions.

### 4.2. AOP Alleviated Oxidative Stress Through Nrf2/Keap1 and TLR4/NF-κB Pathway

Based on our results, we hypothesize that these beneficial effects may be related to the regulation of the Nrf2/Keap1 and TLR4/NF-κB signaling pathways by AOP. To further validate this hypothesis, firstly, we performed in vitro lymphocyte culture experiments using the Nrf2 inhibitor (ML385). Our study found that LPS significantly increased the MDA, 8-OHdG, ROS, and PC levels in the culture medium of PBLs and significantly decreased the T-AOC, SOD, GPx, and CAT activities. LPS also reduced the gene expression of *Nrf2* and its downstream target genes (*SOD*, *GPx*, and *CAT*) while promoting *Keap1* overexpression. In contrast, the LPS + AOP-treated PBLs mitigated these effects by decreasing the oxidative stress markers, increasing the antioxidant enzyme activities, and enhancing the gene expression of *Nrf2* and its downstream targets. Moreover, the overexpression of *Keap1* was suppressed, indicating that AOP alleviates oxidative damage in broiler PBLs through activation of the Nrf2/Keap1 signaling pathway. Further experiments revealed that compared to the AOP + LPS group, the AOP + LPS + ML385 group exhibited decreased *Nrf2* and its downstream target gene expression, increased *Keap1* expression, reduced T-AOC, and lowered the activities of SOD and GPx, along with elevating the MDA and ROS levels in the PBLs. This suggests that blocking the Nrf2/Keap1 pathway with ML385 impedes the mitigating effects of AOP, thus confirming our hypothesis. ML385 interacts directly with the Nrf2 protein, binding to the Neh1-binding region and preventing the Nrf2–MAFG complex from binding to the ARE sequence on the promoter, thereby reducing the transcriptional activity [27].

Moreover, the present study revealed that under stress conditions, the LPS group significantly increased the related gene expression of the NF-κB signaling pathway compared to the control and AOP groups. However, the gene expression of these factors in the LPS + ML385 treatment group remained significantly elevated, indicating that blocking the Nrf2/Keap1 signaling pathway does not affect the expression of the NF-κB signaling pathway. In contrast, both the AOP + LPS and LPS + ML385 + AOP treatment groups alleviated the overactivation of NF-κB caused by LPS but did not mitigate the oxidative damage in the PBLs. This suggests that the Nrf2/Keap1 signaling pathway may function downstream of the TLR4/NF-κB signaling pathway, mediating the antioxidant effects of AOP through their interaction. Studied have shown that Nrf2 activation can suppress NF-κB activity, indicating a downstream effect [28,29]. This elucidates the present results further, showing that AOP did not mitigate the oxidative damage in the PBLs despite inhibiting NF-κB overexpression. This is attributed to the Nrf2 blockade downstream and the subsequent reduction in antioxidant enzyme activity. To further elucidate the interplay between the Nrf2/Keap1 and TLR4/NF-κB signaling pathways, we investigated the effect of AOP on the Nrf2/Keap1 pathway while blocking the TLR4/NF-κB pathway. It is known that LPS activates NF-κB and its target genes through the TLR4 signaling pathway, leading to the generation and release of large amounts of ROS [30], a finding confirmed in this study. Under stress, the LPS group significantly increased the levels of MDA, 8-OHdG, ROS, and PC in the culture medium of the PBLs while significantly decreasing the T-AOC and the activities of SOD, GPx, and CAT. This was accompanied by a marked decrease in the gene expression of *Nrf2* and its downstream targets (SOD, GPx, and CAT), along with increased expression of *Keap1*. Conversely, the addition of AOP (LPS + AOP), PDTC (LPS + PDTC), or both (LPS + AOP + PDTC) to the LPS treatment alleviated these effects. These treatment groups exhibited significant reductions in the MDA, 8-OHdG, ROS, and PC levels, and increased gene expression of factors related to the Nrf2/Keap1 signaling pathway. This suggests that inhibiting NF-κB overactivation can alleviate the excessive decrease in *Nrf2* and its downstream targets caused by LPS and mitigate the overexpression of *Keap1*. It has been shown that overexpression of *IκB*, a negative regulator of the NF-κB pathway, can activate the Nrf2 pathway, while p65 reduces the DNA-binding activity of Nrf2 [31,32,33]. In the present study, AOP significantly alleviated the reduction of *IkBα* in the broiler PBLs caused by LPS. This supports the hypothesis that NF-κB p65 may be located upstream of Nrf2/Keap1 and negatively regulate *Nrf2* expression through feedback mechanisms. The mechanism by which AOP alleviates oxidative stress through the Nrf2/Keap1 and NF-κB pathways requires further in-depth investigation.

Additionally, our study, using ML358, found that AOP can alleviate oxidative stress by activating the Nrf2/Keap1 signaling pathway under non-stress conditions. In the AOP + ML385 group, ML385 inhibited the effects challenged by AOP, and the addition of ML385 alone did not result in significant changes in any of the parameters compared to the control group, suggesting that ML385 itself does not affect the cells. This further indicates that AOP exerts its antioxidative function by activating the Nrf2/Keap1 pathway under non-stress conditions. Meanwhile, in our study, using PDTC showed that under non-stress conditions, the AOP-treated group exhibited increased expression of the TLR4, MyD88, and NF-κB P65 genes. These findings suggest that AOP can activate the Nrf2/Keap1 signaling pathway, potentially not only directly but also by stimulating the TLR4/NF-κB pathway to produce ROS, which in turn activates Nrf2/Keap1 signaling. In contrast, when the TLR4/NF-κB signaling pathway was blocked under non-stress conditions, the antioxidant enzyme activities in the PBLs decreased. This indicates that PDTC inhibited AOP’s activation of NF-κB and its target genes through the TLR4 signaling pathway, leading to reduced ROS generation and, consequently, diminished antioxidant enzyme activities in the PBLs. Collectively, under non-stress conditions, AOP exhibited significant antioxidant activity by activating the Nrf2/Keap1 and TLR4/NF-κB signaling pathways.

## 5. Conclusions

AOP had significant antioxidation activity in vitro, while 150 μg/mL AOP had significant antioxidation activity and could alleviate the oxidative stress caused by LPS via upregulating Nrf2/Keap1 pathway-related gene expression and suppressing TLR4/NF-κB pathway-related gene expression.

## Data Availability

The data presented in this study are available on request from the corresponding author.

## References

[1] Zhang S. (2023). From Challenge to Opportunity: Addressing Oxidative Stress in Animal Husbandry. Antioxidants.

[2] Cheng M., McCarl B., Fei C. (2022). Climate Change and Livestock Production: A Literature Review. Atmosphere.

[3] Juan C.A., Pérez de la Lastra J.M., Plou F.J., Pérez-Lebeña E. (2021). The Chemistry of Reactive Oxygen Species (ROS) Revisited: Outlining Their Role in Biological Macromolecules (DNA, Lipids and Proteins) and Induced Pathologies. Int. J. Mol. Sci..

[4] Gusti A.M.T., Qusti S.Y., Alshammari E.M., Toraih E.A., Fawzy M.S. (2021). Antioxidants-Related Superoxide Dismutase (SOD), Catalase (CAT), Glutathione Peroxidase (GPX), Glutathione-S-Transferase (GST), and Nitric Oxide Synthase (NOS) Gene Variants Analysis in an Obese Population: A Preliminary Case-Control Study. Antioxidants.

[5] Xiao J.L., Liu H.Y., Sun C.C., Tang C.F. (2024). Regulation of Keap1-Nrf2 signaling in health and diseases. Mol. Biol. Rep..

[6] Wang H., Pan L., Si L., Ji R.W., Cao Y.H. (2021). Effects of Nrf2-Keap1 signaling pathway on antioxidant defense system and oxidative damage in the clams Ruditapes philippinarum exposure to PAHs. Environ. Sci. Pollut. Res..

[7] Yue J., Guo P., Jin Y., Li M., Hu X., Wang W., Wei X., Qi S. (2023). Momordica charantia polysaccharide ameliorates D-galactose-induced aging through the Nrf2/β-Catenin signaling pathway. Metab. Brain Dis..

[8] Fu Y.Y., Jiao H.X., Sun J.Z., Obinwanne Okoye C., Zhang H.X., Li Y., Lu X.C., Wang Q.Q., Liu J. (2024). Structure-activity relationships of bioactive polysaccharides extracted from macroalgae towards biomedical application: A review. Carbohyd. Polym..

[9] Yang Q., Chang S.L., Tian Y.M., Li W., Ren J.L. (2024). Glucan polysaccharides isolated from Lactarius hatsudake Tanaka mushroom: Structural characterization and in vitro bioactivities. Carbohyd. Polym..

[10] Wen C., Wu J.P., Zhao X.Y., Li Z.X., Jie Y., Shao T.L., Hou X.F., Zhou L.T., Wang C.F., Wang G.D. (2024). Structural elucidation of an active polysaccharide from Radix Puerariae lobatae and its protection against acute alcoholic liver disease. Carbohyd. Polym..

[11] Pang Y.R., Peng Z.G., Ding K. (2024). An in-depth review: Unraveling the extraction, structure, bio-functionalities, target molecules, and applications of pectic polysaccharides. Carbohyd. Polym..

[12] Trendafilova A., Moujir L.M., Sousa P.M.C., Seca A.M.L. (2021). Research Advances on Health Effects of Edible Artemisia Species and Some Sesquiterpene Lactones Constituents. Foods.

[13] Xiao B., Wang J.H., Zhou C.Y., Chen J.M., Zhang N., Zhao N., Han X.Y., Niu Y.X., Feng Y.B., Du G.H. (2020). Ethno-medicinal study of Artemisia ordosica Krasch. (traditional Chinese/Mongolian medicine) extracts for the treatment of allergic rhinitis and nasosinusitis. J. Ethnopharmacol..

[14] Niu Y., She Z., Su C., Zhao Q., Wang S., Xiao B. (2022). The effects and the mechanisms of naringenin from Artemisia ordosica Krasch on allergic rhinitis based on mast cell degranulation model and network pharmacology. J. Pharm. Pharmacol..

[15] Xing Y.Y., Zheng Y.K., Yang S., Zhang L.H., Guo S.W., Shi L.L., Xu Y.Q., Jin X., Yan S.M., Shi B.L. (2022). Artemisia ordosica Polysaccharide Alleviated Lipopolysaccharide-induced Oxidative Stress of Broilers via Nrf2/Keap1 and TLR4/NF-κB Pathway. Ecotox Environ. Safe.

[16] Xing Y.Y., Zheng Y.K., Yang S., Zhang L.H., Guo S.W., Shi L.L., Xu Y.Q., Jin X., Yan S.M., Shi B.L. (2023). Artemisia ordosica polysaccharide ameliorated LPS-induced growth inhibition and intestinal injury in broilers through enhancing immune-regulation and antioxidant capacity. J. Nutr. Biochem..

[17] Du H.D., Xing Y.Y., Xu Y.Q., Jin X., Yan S.M., Shi B.L. (2023). Dietary Artemisia Ordosica Polysaccharide Enhances Spleen and Intestinal Immune Response of Broiler Chickens. Biology.

[18] Zhu X., Han X., Wang J. (2024). Sufentanil-induced Nrf2 protein ameliorates cerebral ischemia-reperfusion injury through suppressing neural ferroptosis. Int. J. Biol. Macromol..

[19] Chen D., Lou Q., Song X.J., Kang F., Liu A., Zheng C., Li Y., Wang D., Qun S., Zhang Z. (2024). Microglia govern the extinction of acute stress-induced anxiety-like behaviors in male mice. Nat. Commun..

[20] Tariq M., Chen R., Yuan H., Liu Y., Wu Y., Wang J., Xia C. (2015). De Novo Transcriptomic Analysis of Peripheral Blood Lymphocytes from the Chinese Goose: Gene Discovery and Immune System Pathway Description. PLoS ONE.

[21] Jiang N., Yang T., Han H., Shui J., Hou M., Wei W., Kumar G., Song L., Ma C., Li X. (2024). Exploring Research Trend and Hotspots on Oxidative Stress in Ischemic Stroke (2001–2022): Insights from Bibliometric. Mol. Neurobiol..

[22] Yue C., Chen J., Hou R., Tian W., Liu K., Wang D., Lu Y., Liu J., Wu Y., Hu Y. (2017). The antioxidant action and mechanism of selenizing Schisandra chinensis polysaccharide in chicken embryo hepatocyte. Int. J. Biol. Macromol..

[23] Ren H., Li K., Min Y., Qiu B., Huang X., Luo J., Qi L., Kang M., Xia P., Qiao H. (2023). Rehmannia glutinosa Polysaccharides: Optimization of the Decolorization Process and Antioxidant and Anti-Inflammatory Effects in LPS-Stimulated Porcine Intestinal Epithelial Cells. Antioxidants.

[24] Yuan D., Li C., Huang Q., Fu X., Dong H. (2023). Current advances in the anti-inflammatory effects and mechanisms of natural polysaccharides. Crit. Rev. Food Sci. Nutr..

[25] Friedman M. (2016). Mushroom Polysaccharides: Chemistry and Antiobesity, Antidiabetes, Anticancer, and Antibiotic Properties in Cells, Rodents, and Humans. Foods.

[26] Liu B., Ma J., Li T., Li P., Yan D., Zhu J., Zhang X. (2024). Advances in the Preparation, Structure and Bioactivity of Polysaccharides from Lycium ruthenicum Murr.: A Review. Foods.

[27] Su H., Yang F., Fu R., Trinh B., Sun N., Liu J., Kumar A., Baglieri J., Siruno J., Le M. (2022). Collagenolysis-dependent DDR1 signalling dictates pancreatic cancer outcome. Nature.

[28] Li S.J., Ruan D.D., Wu W.Z., Wu M., Wu Q.Y., Wang H.L., Ji Y.Y., Zhang Y.P., Lin X.F., Fang Z.T. (2023). Potential regulatory role of the Nrf2/HMGB1/TLR4/NF-κB signaling pathway in lupus nephritis. Pediatr. Rheumatol..

[29] Zhao L., Zhang Z., Li P., Gao Y., Shi Y. (2024). Bakuchiol regulates TLR4/MyD88/NF-κB and Keap1/Nrf2/HO-1 pathways to protect against LPS-induced acute lung injury in vitro and in vivo. Naunyn Schmiedebergs Arch. Pharmacol..

[30] Kim H.J., Kim H., Lee J.H., Hwangbo C. (2023). Toll-like receptor 4 (TLR4): New insight immune and aging. Immun. Ageing.

[31] Saha S., Buttari B., Panieri E., Profumo E., Saso L. (2020). An Overview of Nrf2 Signaling Pathway and Its Role in Inflammation. Molecules.

[32] Wang R., Perez V.I., Deng H. (2020). Molecular Mechanisms of Nrf2 in Inflammation: Interactions between Nrf2 and Inflammatory Mediators. Nrf2 and its Modulation in Inflammation.

[33] Ando M., Magi S., Seki M., Suzuki Y., Kasukawa T., Lefaudeux D., Hoffmann A., Okada M. (2021). IκBα is required for full transcriptional induction of some NFκB-regulated genes in response to TNF in MCF-7 cells. NPJ Syst. Biol. Appl..

